# The Efficacy and Safety of Autologous Blood Patch for Persistent Air Leaks: A Systematic Review and Meta-Analysis

**DOI:** 10.7759/cureus.36466

**Published:** 2023-03-21

**Authors:** Zaryab Umar, Mahmoud Nassar, Salman Ashfaq, Allison Foster, Jasmine K Sandhu, Jonathan Ariyaratnam, Ricardo Lopez, Theo Trandafirescu

**Affiliations:** 1 Internal Medicine, Icahn School of Medicine at Mount Sinai, Queens Hospital Center, New York, USA; 2 Internal Medicine, Allama Iqbal Medical College, Lahore, PAK; 3 Medicine, Queens Hospital Center, New York, USA; 4 Medicine, Icahn School of Medicine at Mount Sinai, Queens Hospital Center, New York, USA

**Keywords:** bronchopleural fistula, alveolar-pleural fistula, pleurodesis, persistent air leak, autologous blood patching

## Abstract

Persistent air leaks (PALs) are associated with prolonged hospital stays, contamination and sustained infection of the pleural space, and significant morbidity. A fistulous tract between the alveoli and the pleural space is referred to as an alveolar-pleural fistula (APF), whereas a fistulous tract between the bronchiole and the pleural space is referred to as a bronchopleural fistula (BPF). There is no consensus on the treatment, and multiple modalities exist for the management of persistent air leak (PAL). Autologous blood patch (ABP) is a relatively safe and inexpensive method that has been used for many years for the treatment of PALs.

We conducted an electronic database search between 08/24/2022 and 08/27/2022 in PubMed, Embase, and Cochrane using keywords. The following keywords were used: "Blood patch" OR "Autologous blood patch" AND "pleurodesis." Our study included all original studies with the prime focus on the etiology of PALs, clinical characteristics, procedural details of ABP, and outcomes of the proposed treatment. The primary outcomes that were the focus of our study were the time to seal the air leak, the time to remove the chest tube after air leak cessation, and the time to discharge from the hospital. To determine the safety of ABP, we also evaluated the procedural outcomes.

Our findings suggest a statistically significant decrease in the time to air leak cessation when compared to the control group (mean difference of -3.75 {95% CI: -5.65 to -1.85; P=0.001}) with considerable heterogeneity of I^2^=85% and P=0.001. However, the difference was not statistically significant when a lower dose of ABP (50 mL) was compared to a higher dose (100 mL) (mean difference of 1.48 {95% CI: -0.07 to 3.02; P=0.06}) and considerable heterogeneity of I^2^=80% and P=0.03. There was no statistically significant difference in the time to discharge when compared to the control group (mean difference of -2.12 {95% CI: -4.83 to 0.59; P=0.13}) and considerable heterogeneity (I^2^=95% and P<0.001).

When compared to the control group, ABP did not provide any statistically significant difference in the risk ratio for infection (1.18 {95% CI: 0.52 to 2.65; P=0.70} and moderate heterogeneity {I^2^=33% and P=0.20}), pain (1.18 {95% CI: 0.52 to 2.65; P=0.70} and moderate heterogeneity {I^2^=33% and P=0.20}), and fever (0.54 {95% CI: 0.27 to 1.10; P=0.09} and no heterogeneity {I^2^=0% and P=0.50}).

Our study concludes that using ABP caused a statistically significant decrease in the time to air leak cessation when compared to the control group. However, the procedure does not provide a statistically significant difference in the time to discharge from the hospital when compared to conservative treatment. Similarly, there was no statistically significant difference in the risk ratio for complications such as infection, pain, and fever when compared to conservative management. More studies need to be conducted to fully understand the efficacy and safety of ABP in the management of PALs.

## Introduction and background

Air leaks occur when air from one cavity of the body enters another cavity, which usually does not contain air. Pneumothorax is the most common example of this phenomenon, in which air escapes from the bronchial tree or alveoli and enters the pleural space through a fistulous tract. Treatment usually consists of observation with spontaneous resolution, insertion of a chest tube with wall suction, and, in severe cases, surgical intervention. When a chest tube is placed, the air leak is identified by bubbling in the chest drainage system. Air leaks that persist for more than 5-7 days are considered persistent air leaks (PALs) [1]. Patients with fistulous tracts are at risk of significant morbidity and mortality due to ventilation/perfusion mismatch and pleural contamination with respiratory flora [2]. Depending on the initial cause of persistent air leaks, a variety of treatment options have been explored. These include prolonged chest tube drainage, chemical pleurodesis, autologous blood patch (ABP) pleurodesis, and temporary unilateral valve placements [1]. Pleurodesis using autologous blood patches has been used to treat persistent air leaks with variable results. In this study, we reviewed the literature on autologous blood patch pleurodesis for PAL and its efficacy in treating the condition.

## Review

Materials and methods

Search Strategy and Study Selection

This study followed Preferred Reporting Items for Systematic Reviews and Meta-Analyses (PRISMA) guidelines for systematic reviews and meta-analyses, which do not require protocol registration [3]. An electronic database search was conducted for relevant studies published from 08/24/2022 to 08/27/2022 in PubMed, Embase, and Cochrane using keywords. We used these search terms in each database: "Blood patch" OR "Autologous blood patch" AND "pleurodesis." This search included all original studies (cohort, cross-sectional, and case-control studies) describing the etiology and duration of air leaks, clinical characteristics, procedures of autologous blood patch with outcome and complications associated with the intervention, and commentaries and case series with more than 10 patients. The exclusion criteria included non-original reports, which were either reviews, letters to editors, or commentaries that did not include patient data; case reports or case series of less than 10 patients; unextractable or irrelevant data; articles not published in English; duplicate records; animal studies; overlapped data; and full texts that were not available, unextractable, or irrelevant data.

The primary outcomes were the time to seal the air leak, the time to remove the chest tube after the air leak was stopped, and the time to discharge from the hospital following the air leak's cessation. A secondary outcome was the percentage of success and complications. In order to ensure that we did not miss any relevant studies, we manually searched the references of our included papers. All original studies that reported persistent air leaks and ABP were included in the study. Our systematic review was screened by two independent reviewers for titles and abstracts, followed by a full-text screening to ensure that relevant papers were included. We resolved disagreements by discussion and by referring them to the senior author when necessary.

Data Extraction

We developed a data extraction sheet using Microsoft Excel (Microsoft® Corp., Redmond, WA). Two independent reviewers extracted data using the Excel sheet. Disagreements and discrepancies were all resolved through discussions with the senior author.

Quality Assessment

The risk of bias in the included studies was evaluated by one independent reviewer. A risk-of-bias assessment tool developed by the National Institutes of Health (NIH) was used to assess the quality of the included studies [4].

Statistical Analysis

A descriptive analysis was conducted using Statistical Package for Social Sciences (SPSS) (IBM SPSS Statistics, Armonk, NY). Review Manager (RevMan) 5 (Cochrane, London, England) was used to develop the forest plot and funnel plot.

Results

Search Results

**Figure 1:**
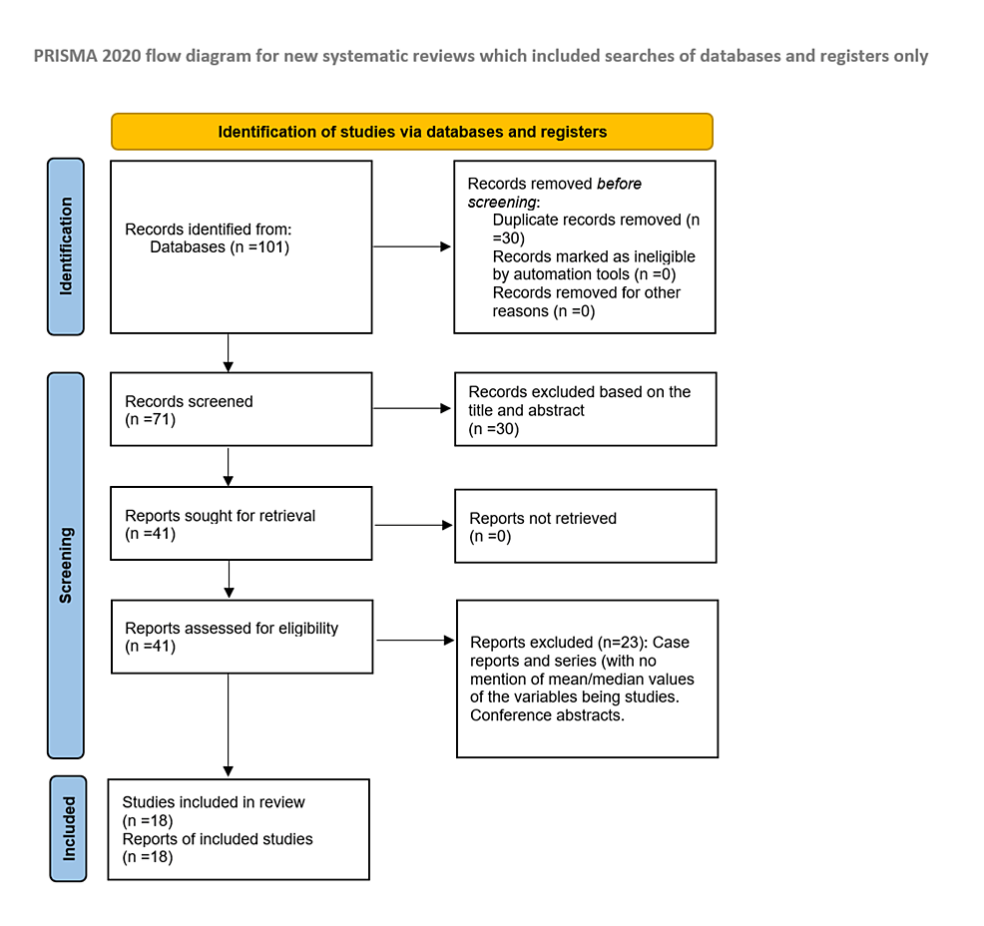
Flow diagram of the PRISMA screening process for this systematic review and meta-analysis PRISMA: Preferred Reporting Items for Systematic Reviews and Meta-Analyses

Following the elimination of 30 duplicate records with EndNote 20 (Clarivate, London, England), we identified 101 records. Based on the title and abstract screening, 41 records were selected for further full-text screening. After excluding 23 papers from the full-text screening phase, 18 studies were included in our study (Figure 1).

Study Characteristics and Quality of the Included Studies

The baseline characteristics of the included studies are summarized in Table 1. There were 18 studies included: six were retrospective [5-10], seven were randomized controlled trials (RCTs) [11-17], one study was a non-randomized controlled trial [18], three were prospective studies [19-21], and one was a case series involving 11 patients with original data [22]. The sample sizes of the included studies ranged from 11 to 167 [22,20]. Patients ranging in age from adults to geriatrics were included in the study. Male patients constituted 77% of the study population.

**Table 1:** Characteristics of patients and autologous blood patch deployment (ABP) in the articles included M, male; F, female; n, number of patients/events; SD, standard deviation; FT, flexible thoracoscopy; IQR: interquartile range; CTD, chest tube drainage; BOS: bronchial occlusion using silicone spigots; ARDS; acute respiratory distress syndrome

First author, year	Publication type	Number of patients, sex (M/F)	Age (years)	Underlying disease (n)	Previous intervention (n)	Duration of air leak before ABP (n)	Size of air leak (mean±SD)	Classification of air leak (type/n)*
Apilioğulları et al., 2020 [5]	Retrospective study	24 (17/7)	Mean±SD: 59.9±12.2	Lung cancer (11), metastatic lung cancer (4), iatrogenic pneumothorax (malignant pleural effusion drainage) (4), bullous lung (6), and chronic disease (3)	Lobectomy (6), wedge resection (1), metastasectomy (4), and bullectomy (4)	>7 days	-	-
Andreetti et al., 2007 [11]	Randomized controlled study	Group A, 12 (7/5); group B, 13 (7/6); group C, 15 (9/6)	Mean±SD: group A, 68.5±6.9; group B, 65.2±6.2; group C, 67±7.1	Adenocarcinoma (19), squamous cell carcinoma (14), and mixed adenosquamous carcinoma (1)	Lobectomy (34)	≥6 days	-	-
Akar et al., 2020 [6]	Retrospective study	Group 1, 20 (17/3); group 2, 22 (19/3)	Mean±SD: 52.1±16.0	Secondary spontaneous pneumothorax (SSP) (42), chronic obstructive pulmonary disease (COPD) (24), tuberculosis (10), interstitial lung disease (ILD) (6), and histiocytosis (2)	Tube thoracostomy (42)	>7 days after thoracostomy	-	-
Ferraroli et al., 2022 [7]	Retrospective study	Study (FT) group, 17 (14/3); control (thoracotomy) group, 6 (6/0)	Median age: study (FT) group, 71 (IQR: 47-82); control (thoracotomy) group, 67 (IQR: 22-75)	Adenocarcinoma (12), squamous carcinoma (4), other carcinomas (7), and others (15)	Lung resection (18) and tube thoracostomy (23)	≥11 days after thoracostomy	-	-
Cao et al., 2012 [12]	Randomized controlled study	Group A, 11 (10/1); group B, 11 (11/0); group C, 11 (11/0); group D, 11 (10/1)	Median (IQR): group A, 65 years (59-73); group B, 65 (56-70); group C, 68 (52-75); group D, 63 (59-69)	COPD leading to a secondary spontaneous pneumothorax (SSP) (44) (stages 3-4)	Tube thoracostomy (44)	≥7 days	Group A, 2±0.7; group B, 2±0.6; group C, 1.9±0.7; group D, 1.9±0.8	Size, 1, 12; size 2, 22; size 3, 10
Zhang et al., 2019 [13]	Multicenter randomized controlled trial	CTD group, 50 (47/3); ABP group, 50 (47/3); BOS group, 50 (45/5)	Mean±SD: group A, 53.24±16.02; group B, 56.42±14.88; group C, 57.44±13.72	SSP (150), chronic obstructive pulmonary disease (65), pulmonary bullae (47), pneumonia (24), pulmonary cancer (20), bronchiectasis (11), asthma (9), and pulmonary fibrosis (6)	Tube thoracostomy (150)	>7 days	Size of pneumothorax (%) by using an automatic calculation program or Rhea's method manually: group A, 52.82±2.08; group B, 54.72±1.84; group C, 56.06±1.69	-
Ibrahim et al., 2019 [14]	Randomized controlled study	Group A, 23 (19/4); group B, 24 (20/4)	Mean±SD: group A, 57.6±10.2; group B, 61.6±10.0	Secondary spontaneous pneumothorax (47)	Tube thoracostomy (47)	≥3 days after chest tube insertion	Group A: size 1, 4; size 2, 10; size 3, 9; fully expanded lung, 7. Group B: size 1, 4; size 2, 12; size 3, 8; fully expanded lung, 8	Size 1, 8; size 2, 22; size 3, 17; fully inflated, 15
Dye et al., 2020 [8]	Retrospective study	19 (11/8): mass resection group, 15; lung volume reduction group, 4	Median: 67.9 (50.3-78.7)	COPD (5) and emphysema (5)	Tube thoracostomy (19)	Median: seven days (IQR: 4-19)	-	Mild, 3; moderate, 12; severe, 4
Aihara et al., 2011 [9]	Retrospective study	34 (25/9): ABP group, 13; chemical pleurodesis group, 10	Median: 64 (IQR: 21-82)	Interstitial lung disease with pneumothorax (34), idiopathic interstitial pneumonia (17), sarcoidosis (6), berylliosis (1), collagen vascular disease (7), chronic hypersensitivity pneumonitis (1), pulmonary Langerhans cell histiocytosis (1), and drug-induced pneumonia (1)	Tube thoracostomy (34)	≥3 days after thoracostomy	-	-
Lang-Lazdunski et al., 2004 [22]	Case series		Mean±SD: 56±15	Adenocarcinoma (6), recurrent pneumothorax (1), primary spontaneous pneumothorax (PSP) (1), bullous emphysema and SSP (1), pulmonary lymphoma (1), Pancoast tumor (1), and large cell carcinoma (1)	Lung resection (11), wedge resection (2), lobectomy (7), thoracotomy/bullectomies+pleurectomy (1), lobectomy+chest wall resection (1), and tube thoracostomy (11)	>7 days		Grade 1: 11
Ando et al., 1999 [19]	Prospective study	11 (10/1)	Median: 70 (22-83)	Emphysema (7), tuberculosis (2), lung cancer (1), and histiocytosis X (1)	Tube thoracostomy (11)	≥6 days after thoracostomy	-	-
Shackcloth et al., 2006 [15]	Randomized controlled trial	20 (13/7): study group, 10 (7/3); control group, 10 (6/4)	Median: study group, 63 (61-72); control group, 64 (53-70)	Underlying disease process (10)	Tube thoracostomy (20) and upper lobectomy (14)	≥5 days	-	Study group: size 1, 2; size 2, 7; size 3, 1. Control group: size 1, 2; size 2, 7; size 3, 1
Khan et al., 2017 [16]	Randomized controlled trial	30 (26/4): study group, 16 (14/2); control group, 14 (12/2)	Average age: study group, 37.69; control group, 29.93	Recurrent primary spontaneous pneumothorax (30)	Tube thoracostomy (30)	(Just written as "persistent air leak," days not mentioned)	-	-
Evman et al., 2016 [10]	Retrospective study	31 (27/4)	Mean±SD: 53.7±18.9	SSP (31), tuberculosis (10), COPD (9), malignancy (7), COPD+tuberculosis (4), and histiocytosis (1)	Tube thoracostomy (31)	3 days	-	-
Martínez-Escobar et al., 2006 [18]	Non-randomized controlled study	54 (24/30): study group, 27 (12/15); control group, 27 (12/15)	Median: study group, 44 (24-73); control group, 44 (19-78)	All with ARDS, pneumothorax, and PAL receiving mechanical ventilation	Tube thoracostomy/mechanical ventilation	-	-	-
Cagirici et al., 1998 [20]	Prospective study	167 (125/42): study group, 32 (29/3); control group, 135 (96/39)	Mean±SD: study group, 45.5±15.9; control group, 39.2±12.8	Primary spontaneous pneumothorax (32) and secondary spontaneous pneumothorax (135)	Tube thoracostomy	Air leak persisting for more than 48 hours after a first episode or 24 hours after a recurrent episode of spontaneous pneumothorax	-	-
Cobanoglu et al., 2009 [21]	Prospective study	50 (32/18): autologous group, 20; talc group, 19; tetracycline group, 11	Median: 39 (14-69)	PSP (19) and SSP (31)	Previous interventions not mentioned	>7 days	-	Autologous blood group: size 1, 5; size 2, 13; size 3, 2. Talc group: size 1, 5; size 2, 11; size 3, 3. Tetracycline group: size 1, 2; size 2, 7; size 3, 2
Narenchandra et al., 2022 [17]	Randomized controlled trial	38 (29/9)	Mean: ABP group, 43.47 (18-74); doxycycline group, 48.84 (19-75)	SSP (38), COPD (20), malignancy (10), COPD with malignancy (6), ILD (7), bulla (6), and cyst (2)	Tube thoracostomy	>3 days	Median air leak grade: ABP group, 2; doxycycline group, 1	-

The Characteristics of the Patient and the Etiology of the Air Leak

Persistent air leaks can result from a variety of clinical pathologies. A study conducted by Apilioğulları et al. reported that lung cancer was the cause of PAL in 11 cases, as well as metastatic lung cancer in four cases [5]. Andreetti et al. report that adenocarcinoma is the most common cause of PAL in their study population, followed by squamous cell carcinoma [11]. Ferraroli et al. reported similar findings, with adenocarcinoma being the most common etiology of PAL [7]. Lang-Lazdunski et al. reported the most common pathology in patients with persistent air leaks to be adenocarcinoma, but lymphoma, large cell carcinoma, and Pancoast tumor have each been reported in one patient [22]. According to Akar et al. [6], Cao et al. [12], and Zhang et al. [13], secondary spontaneous pneumothorax is the primary cause of PAL.

In a few studies, primary and secondary pneumothorax have been identified as the etiology of PALs, but the underlying cause of secondary pneumothorax has not been described [14,16,20,21]. Interstitial lung disease (ILD) and tuberculosis were also suggested as the probable causes of PAL in some studies reported [6,9,10,17,19]. A minimum duration of air leaks of 2-11 days was required for study participants to be eligible for ABP. A median of seven days has been reported by Dye et al. [8], and the range is from four to nine days. A wide range of interventions was performed in the study subjects prior to ABP, the most common of which was tube thoracostomy (since the intervention was necessary to manage an air leak that caused a pneumothorax to develop).

Procedural Details

Apilioğulları et al. [5], Cao et al. [12], and Khan et al. [16] conducted ABP with 1-2 mL/kg, 0.5-2 mL/kg, and 1 mL/kg of blood, respectively. Andreetti et al. reported a study design where 50 mL of autologous blood was infused into group A participants and 100 mL of blood was infused into group B participants, with group C serving as an observational group [11]. In a similar study design, Akar et al. infused 60 mL of blood into participants in group A and 120 mL into participants in group B [6]. Ferraroli et al. [7], Zhang et al. [13], Shackcloth et al. [15], and Martínez-Escobar et al. [18] performed studies in which the experimental groups received 10 mL, 20-30 mL, 120 mL, and 50-75 mL of autologous blood, respectively. Dye et al. conducted a retrospective study in which subjects receiving lung mass resection received 45-120 mL and 140 mL in their first and second attempts. During the first and second attempts, the lung volume reduction group received 75-120 mL and 80-110 mL, respectively. In the remaining studies, patients in experimental groups received 50 mL of autologous blood [8].

Outcomes, Complications, and Results of the Meta-Analysis

Our meta-analysis included eight studies [6,11,13-16,18,20]. This meta-analysis focused on the time to seal air leaks (days) and the time to discharge from the hospital (days). This meta-analysis focused on complications related to fever, infection, and pain. The time to seal an air leak (days) was compared between the experimental group receiving 50 mL and the control group.

**Figure 2:**
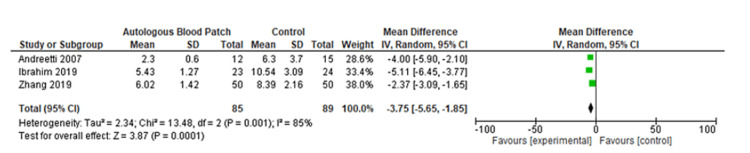
Forest plot analysis of the time to seal air leak (days), 50 mL blood instillation compared to the control group [11,13,14]

**Figure 3:**
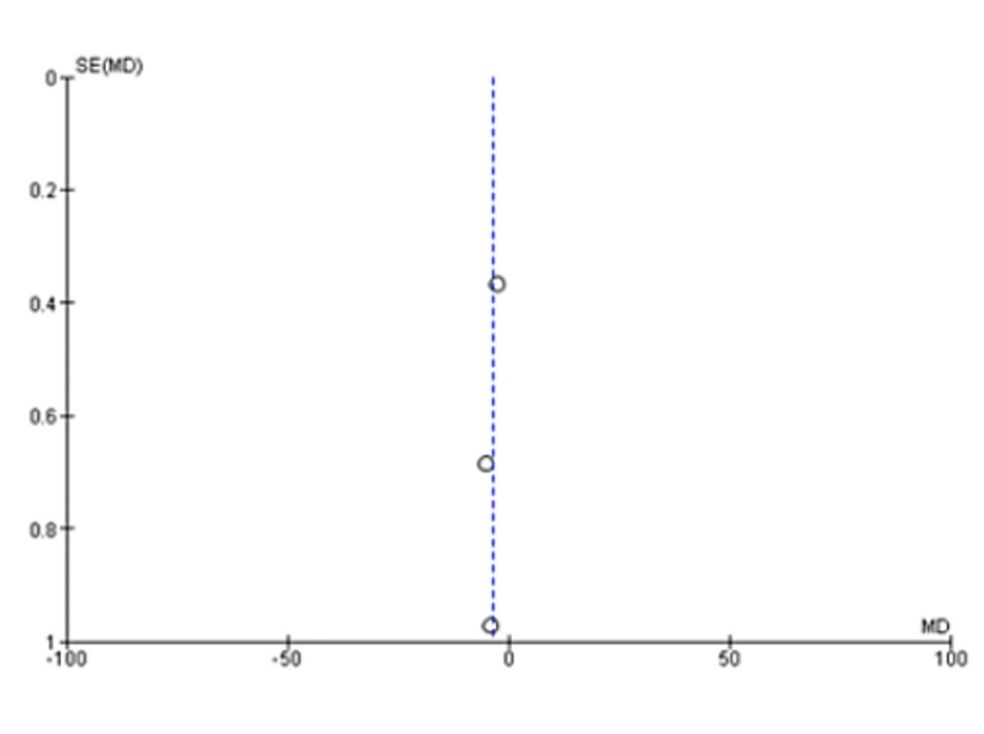
Funnel plot analysis of the time to seal air leak (days), 50 mL blood instillation compared to the control group [11,13,14]

**Figure 4:**
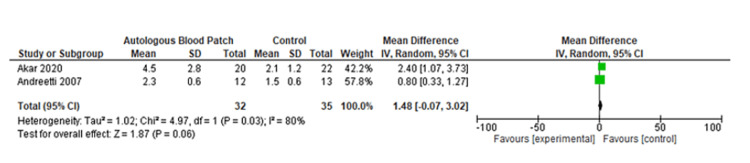
Forest plot analysis of the time to seal air leak (days), comparison of 50 mL and 100 mL blood instillation group with 60 mL and 120 mL blood instillation group [6,11]

**Figure 5:**
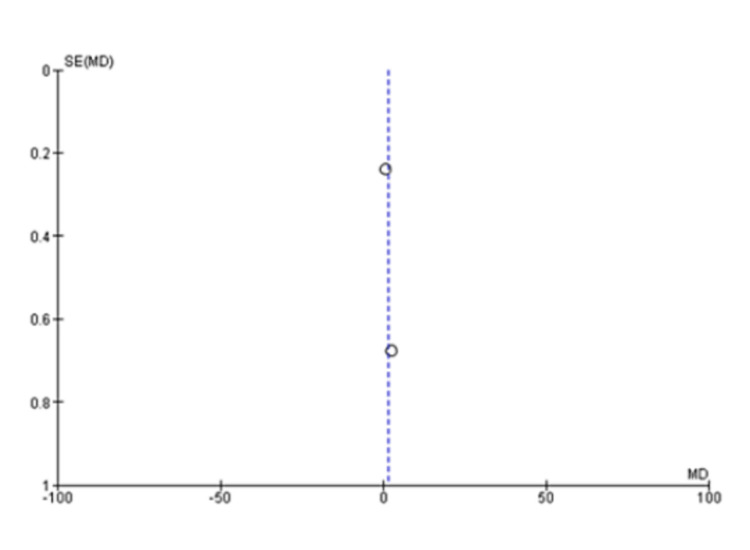
Funnel plot analysis of the time to seal air leak (days), comparison of 50 mL and 100 mL blood instillation group with 60 mL and 120 mL blood instillation group [6,11]

The pooled analysis from three studies includes 174 individuals. Based on this analysis, the mean difference for the autologous blood patch was -3.75 (95% CI: -5.65 to -1.85; P=0.001) with considerable heterogeneity of I^2^=85% and P=0.001 (Figure 2 and Figure 3) [11,13,14]. An analysis of pooled data from two studies comparing a high dose (100 mL) of autologous blood with a lower dose (50 mL) that included 67 patients was conducted. Statistical analysis revealed no significant differences between the two groups, with a mean difference of 1.48 (95% CI: -0.07 to 3.02; P=0.06) and considerable heterogeneity of I^2^=80% (P=0.03) (Figure 4 and Figure 5) [6,11].

**Figure 6:**
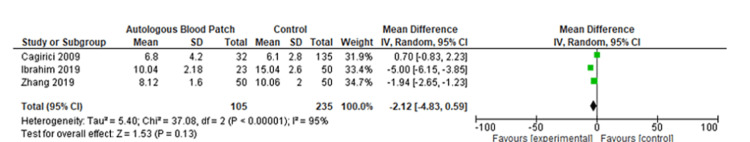
Forest plot analysis of the time to discharge [13,14,20]

**Figure 7:**
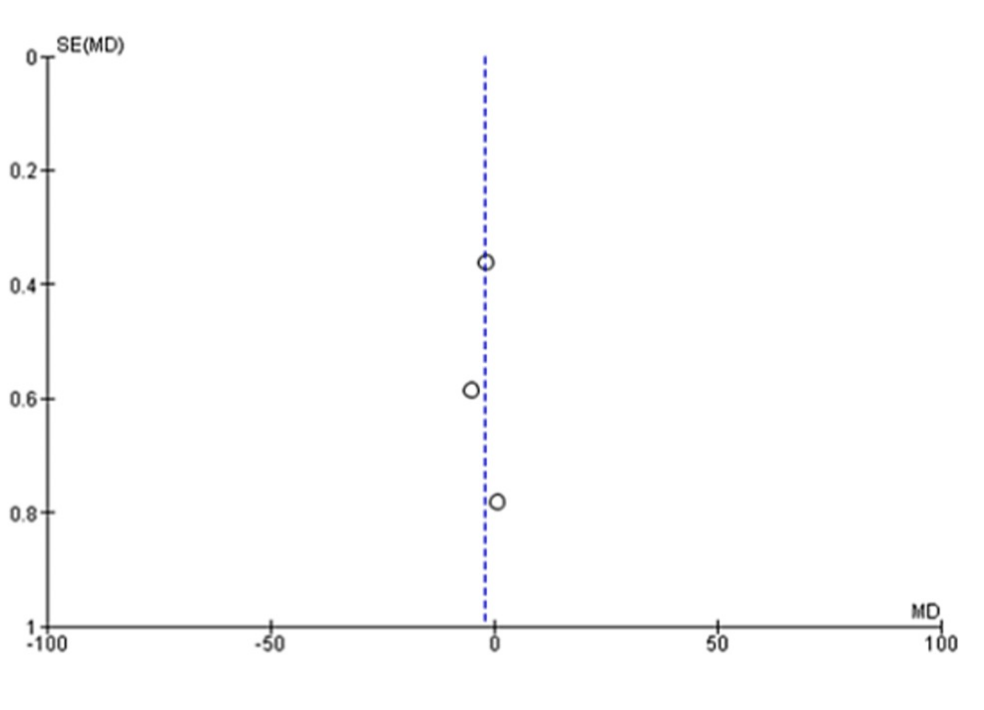
Funnel plot analysis of the time to discharge [13,14,20]

**Figure 8:**
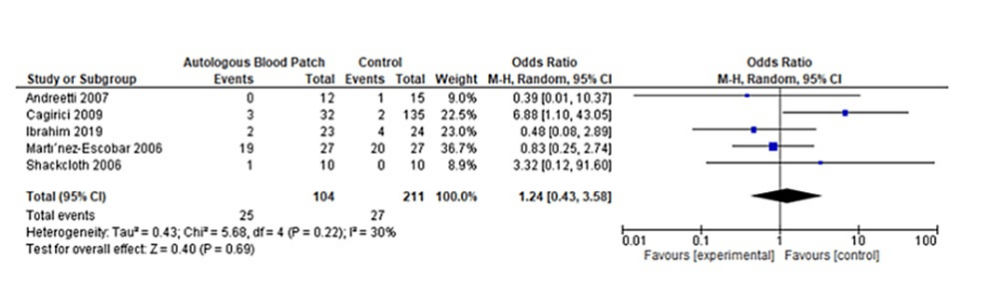
Forest plot analysis of infection [11,14,15,18,20]

**Figure 9:**
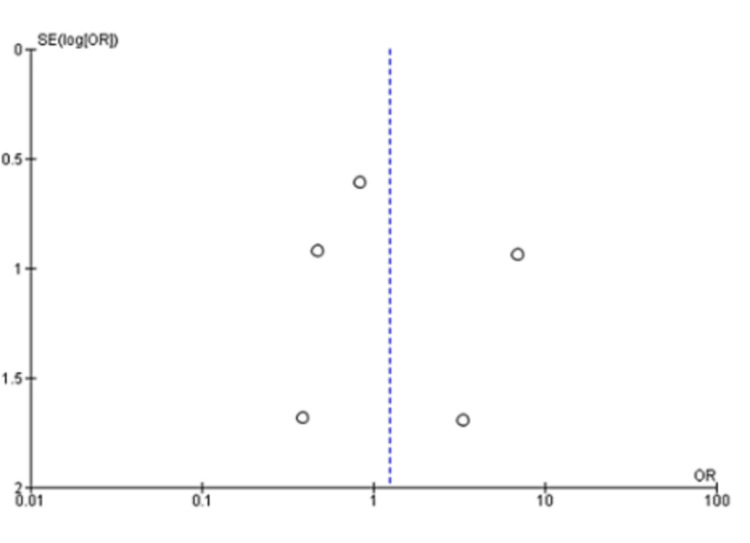
Funnel plot analysis of infection [11,14,15,18,20]

**Figure 10:**
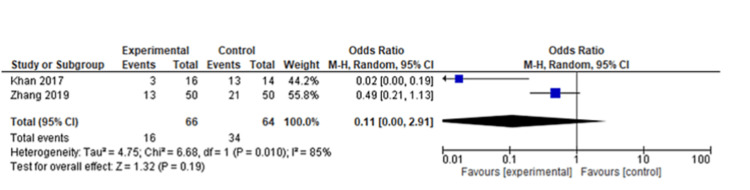
Forest plot analysis of pain [13,16]

**Figure 11:**
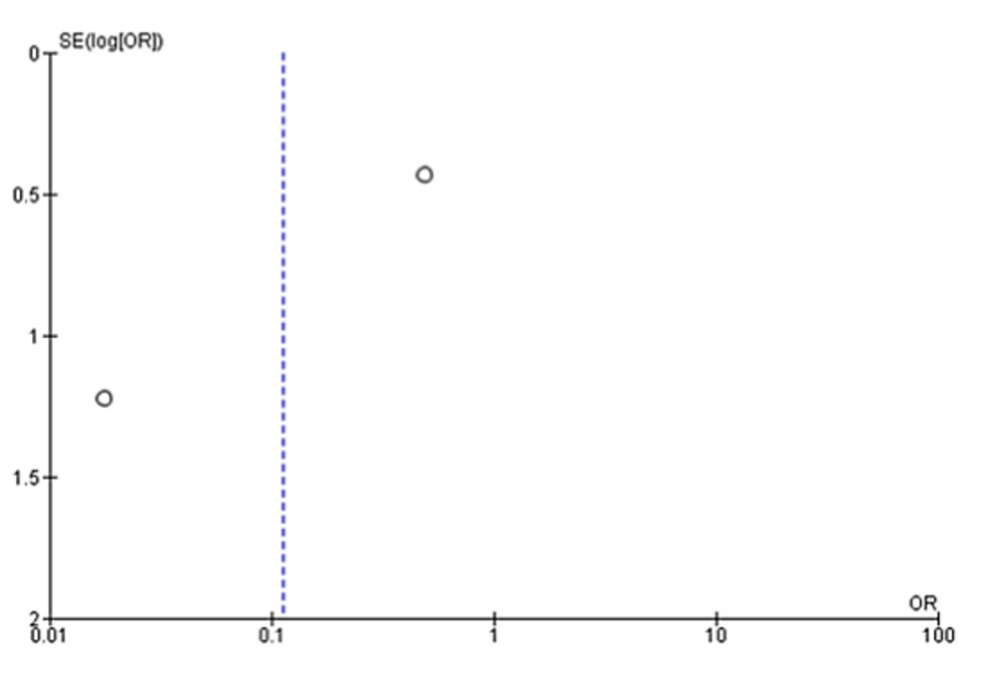
Funnel plot analysis of pain [13,16]

**Figure 12:**
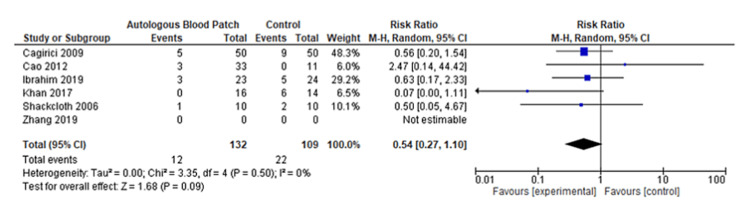
Forest plot analysis of the development of fever [11-16]

**Figure 13:**
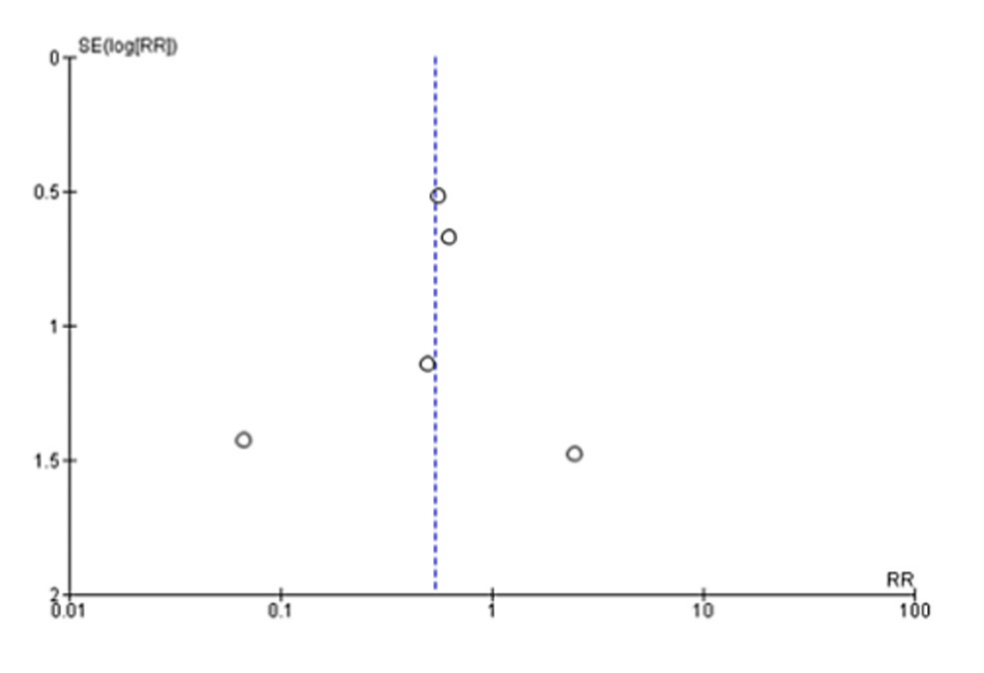
Funnel plot analysis of the development of fever [11-16]

A pooled analysis of the time to discharge included three studies with 340 patients that showed no significant differences between two groups of patients with a mean difference of -2.12 (95% CI: -4.83 to 0.59; P=0.13) and considerable heterogeneity (I^2^=95% and P<0.001) (Figure 6 and Figure 7) [13,14,20]. Based on a pooled analysis of five studies including 315 patients for the incidence of infection, there was no significant difference with a risk ratio of 1.18 (95% CI: 0.52 to 2.65; P=0.70) and moderate heterogeneity (I^2^=33% and P=0.20) (Figure 8 and Figure 9) [11,14,15,18,20]. A pooled analysis of two studies for pain included 130 patients with nonsignificant differences, with a risk ratio of 0.38 (95% CI: 0.13 to 1.15; P=0.09) and moderate heterogeneity of I^2^=72% and P=0.09 (Figure 10 and Figure 11) [13,16]. The pooled analysis for the development of fever included six studies with 241 patients that showed no significant difference with a risk ratio of 0.54 (95% CI: 0.27 to 1.10; P=0.09) and no heterogeneity (I^2^=0% and P=0.50) (Figure 12 and Figure 13) [11-16].

Risk of Bias of Included Studies

Based on the NIH tool, all of the studies included scored at least a 10 on the assessment. We evaluated the risk of bias of the case series included in our study using the NIH risk assessment tool (Table 2) [4].

**Table 2:** Outcomes of autologous blood patch deployed in the articles selected n, number of patients; SD, standard deviation; ICU, intensive care unit; AFib, atrial fibrillation; IQR, interquartile range; FT, flexible thoracoscopy; IQR, interquartile range; CTD, chest tube drainage; BOS, bronchial occlusion using silicone spigots; ABP, autologous blood patch; ARDS, acute respiratory distress syndrome

First author, year	Blood instilled	Intervention in control group	Time to seal air leak (days)	Time to remove the chest tube after cessation of air leak (days)	Time to discharge from hospital after cessation of air leak (days)	Number of instillations: 0, 1, 2, 3, and 4	Percentage of success	Complications (n)
Apilioğulları et al., 2020 [5]	1-2 mL/kg (50-70 mL)	No control group	Within two days (seven patients)	-	-	0, 20, 4, 0, and 0	Lung expansion was achieved in 17 patients during the follow-up period	-
Andreetti et al., 2007 [11]	mL: group A, 50; group B, 100	Group C: observation	Mean±SD: group A, 2.3±0.6; group B, 1.5±0.6; group C, 6.3±3.7	Group A, 1; group B, 1; group C, 1	Group A, 2; group B, 2; group C, 2	Group A: 0, 12, 0, 0, and 0. Group B: 0, 13, 0, 0, and 0	-	Pneumonia, 1 (group C); atrial fibrillation, 2 (group C)
Akar et al., 2020 [6]	Group 1, 60 mL; group 2, 120 mL	Both groups received autologous blood	Group 1, 4.5±2.8; group 2, 2.1±1.2	Group 1, 7.5±3.3; group 2, 3.1±1.3 (not mentioned in terms of days after air leak cessation)	Hospitalization duration (not mentioned in terms of days after air leak cessation): group 1, 9.6±3.6; group 2, 4.7±1.2	Group 1: 0, 20, 12, 4, and 4. Group 2: 0, 22, 20, 2, and 0	Overall success rate: 100% in terms of air leak cessation within two days. Group 1, 20%; group 2, 90.9%	Group 1: pain (2). Group 2: pain (6) and fever (3)
Ferraroli et al., 2022 [7]	mL: 10	Thoracotomy	-	Median time: FT group, 10 (2-31); thoracotomy group, 7 (3-73)	In-hospital stay median time: FT group, 24 (IQR: 10-53); thoracotomy group: 23 (IQR: 17-85)	Study group: 0, 17, 0, 0, and 0	-	-
Cao et al., 2012 [12]	mL/kg: group A, 0.5; group B, 1; group C, 2	Group D: saline (1 mL/kg)	Group A, 10.9; group B, 6.5; group C, 6.2; group D, 11.4	Group A, 2; group B, 2; group C, 2; group D, 2	-	According to the size of the air leak: Size 1: 0, 4, 4, 3, and 0. Size 2: 0, 3, 5, 8, and 0. Size 3: 0, 0, 0, 5, and 0	At day 20th: size 1, 92; size 2, 73; size 3, 50	Fever, 3 (group B, 1; group C, 2); pleural effusion, 4 (group B, 2; group C, 2)
Zhang et al., 2019 [13]	20-30 mL	CTD group: chest tube-attached water seal drainage. BOS group: bronchial occlusion using silicone spigots	CTD group, 8.39±2.16; ABP group, 6.02±1.42; BOS group, 5.21±1.66	_	CTD group, 10.06±2.00; ABP group, 8.12±1.60; BOS group, 7.31±1.62	ABP group: 0, 50, 0, 0, and 0	At day 14: CTD group, 60%; ABP group, 82%; BOS group, 84%	Fever (CTD group, 9; ABP group, 5; BOS group, 8), pain (CTD group, 21; ABP group, 13; BOS group, 18), cough (CTD group, 18; ABP group, 14; BOS group, 22), hemoptysis (CTD group, 6; ABP group, 50; BOS group, 50), and spigot displacement in four patients of BOS group
Ibrahim et al., 2019 [14]	mL: 50	Group B: conservative management=observation, bronchodilators, and physiotherapy group	Mean±SD: group A, 5.43±1.27; group B, 10.54±3.09	Mean±SD: group A, 7.87±2.03; group B, 12.79±2.81	Mean±SD: group A, 10.04±2.18; group B, 15.04±2.60	Group A: 0, 6, 12, 5, and 0. Group B: 8, 6, 9, 1, and 0	Within seven days: group A, 78.3%; group B, 8.33%	Fever, 8 (group A, 3; group B, 5); pleural infection, 6 (group A, 2; group B, 4)
Dye et al., 2020 [8]	Mass resection group: first attempt, 45-120 mL; second attempt, 140 mL. Lung volume reduction group: first attempt, 75-120 mL; second attempt, 80-110 mL	-	Median time (in hours): mass resection group, 1 (IQR: 1-14); lung volume reduction group, 2 (IQR: 1-9)	-	-	Mass resection group: 0, 11, 3, 1, and 0. Lung volume reduction group: 0, 1, 3, 0, and 0	Overall, 94.8%; mass resection group, 93.3%; lung volume reduction group, 100%	Mass resection group: empyema (4), AFib, lung transplantation, and persistent tachycardia in three patients. Lung volume reduction group: AFib, lung transplantation, and persistent tachycardia in two patients
Aihara et al., 2011 [9]	mL: 50	Chemical pleurodesis with minocycline (Minomycin) 200 mg in 50 mL isotonic saline or OK-432 (picibanil) 10 KE in 50 mL isotonic saline	Air leak seal within two days: ABP group, 16; chemical pleurodesis group, 11	-	-	Total number of blood instillations in ABP group: 22	Group A, 72.7%; group B, 78.6%	ABP group, none; chemical pleurodesis group, 2; respiratory complications, one case each of acute exacerbation of idiopathic pulmonary fibrosis and asthma attack
Lang-Lazdunski et al., 2004 [22]	mL: 50	-	Within two days: 11	0.5 days	Median in-hospital stay: nine (IQR: 7-16) days	0, 11, 0, 0, and 0	100%	Pneumonia (1), fever (2), pleural fluid *Staphylococcus aureus* growth (1), and *Staphylococcus epidermidis* growth (1)
Ando et al., 1999 [19]	mL: 50	-	-	2 days after the air leak stopped	-	Results given as 17 occurrences for inflated and deflated lungs: Inflated lung: first instillation, 6 (two of them failed); second instillation, 4 (two of them failed). Deflated lung: first instillation, 3 (two of them failed); second instillation, 4 (one of them failed)	Persistent air leak was resolved in 10 instances (59%). Six were from 10 instances of inflated lung (60%), and four were from seven instances with deflated lungs (57%)	Recurrence: after eight days (1) and five months (1)
Shackcloth et al., 2006 [15]	mL: 120	Tube thoracostomy alone (eight of the 10 patients still had an air leak by the 10th postoperative day and therefore crossed over to receive a total of 15 intrapleural blood treatments)	Median time from first pleurodesis: study group, 1 (IQR: 1-3); control group, 3 (IQR: 1-4)	Median time: study group, 6.5 (IQR: 6-8); control group, 12 (IQR: 11-13)	Median: study group, 8 (IQR: 7-9); control group, 13.5 (IQR: 12-14)	Study group: 0, 7, 2, 1, and 0. Control group: 2, 3, 3, 2, and 0	By next day: overall success rate on 17 of the 29 occasions (58.6%). Study group: nine of 14 occasions (64.3%). Control group: eight of 15 (53.3%)	Study group: fever and fluid collection that grew* Staphylococcus aureus* (1). Control group: fever (2)
Khan et al., 2017 [16]	Average: 54.7 mL/kg=1 mL/kg body weight	Bleomycin, 1 IU/kg body weight; average, 55.7 mL in 100 mL normal saline	Study group, one day (9) and two days (4); control group, one day (7) and two days (6)	-	-	Study group: 0, 16, 0, 0, and 0	Success rate: study group, 93.7%; control group, 100%	Study group: pain (3) and recurrence (3 after on average of 11.77-month follow-up). Control group: pain (13), fever (6), and recurrence (3 after on average of 11.77-month follow-up)
Evman et al., 2016 [10]	50 mL	-	1 day (29 patents) and 40 hours (one patient)	1 day	1 day after pleurodesis	0, 29, 1, 0, and 0	Within one day: 93.5%	Recurrence (4)
Martínez-Escobar, 2006 [18]	50-75 mL	Intercostal drainage tube with water seal	-	Seal time in terms of mean (range): study group, 2 (1-5); control group, 10 (4-16). Weaning time in terms of mean (range): study group, 5 (2-12); control group, 16 (8-26)	ICU stay time in terms of mean (range): study group, 16 (8-62); control group, 29 (16-47)	Study group: 0, 27, 0, 0, and 0	-	Study group: fever (3.4% of the cases), respiratory tract infection (19), acute renal failure (12), hemodynamic instability (7), other alterations (13), and death (1). Control group: respiratory tract infection (20), acute renal failure (15), hemodynamic instability (15), other alterations (19), and death (8)
Cagirici et al., 1998 [20]	mL: 50	Simple drainage, with or without suction	Study group: two days (25) and three days (2)	Duration of tube drainage (mean±SD) in hours: study group, 75.0±22.8; control group, 94.1±48.8	Hospital stay (mean±SD) in days: study group, 6.8±4.2; control group, 6.1±2.8	Study group: 0, 32, 0, 0, and 0	84% in the study group at the end of three days	Study group: minor pleural effusion (5), fever (4), major empyema (3), and persistent air leak (2). Control group: the recurrence of spontaneous pneumothorax during 12-48-month follow-up period (22) and empyema (2)
Cobanoglu et al., 2009 [21]	mL: 50	Talc group, 5 g of sterile talc powder in 40 mL saline and 10 mL prilocaine; tetracycline group, 250 mg lidocaine and 20 mg/kg tetracycline in 150 mL saline	0.5 (1/2) days: autologous blood group, 4; talc group, 2; tetracycline group, 0. One day: autologous blood group, 7; talc group, 5; tetracycline group, 0. Two days: autologous blood group, 2; talc group, 7; tetracycline group, 2 >3 days: autologous blood group, 2; talc group, 2; tetracycline group: 5	-	-	Autologous blood group: 0, 20, 0, 0, and 0	Autologous blood group, 75.0%; talc group, 84.2%; tetracycline group, 63.6%	Autologous blood group: empyema (1). Talc group: fever (12), pain (8), emesis-vomiting (4), hypotension (3), supraventricular tachycardia (1), convulsion (1), dyspnea (11), and ARDS (1). Tetracycline group: fever (9), pain (10), dyspnea (4), elevated liver enzymes (5), and empyema (1)
Narenchandra et al., 2022 [17]	mL: 50	200 mg of 2% lignocaine and doxycycline 500 mg mixed with 50 mL normal saline solution	Median time (in hours): ABP group, 24 hours (IQR: 12-24); doxycycline group, 36 hours (IQR: 24-72)	-	-	ABP group: 0, 19, 0, 0, and 0	At the end of seven days: ABP group, 94.7%; doxycycline group, 84.2%	ABP group: pain (3), fever (2), giddiness and chills (1), and recurrence within 28 days (1). Doxycycline group: pain (14), fever (7), giddiness and chills (1), and recurrence within 28 days (1)

Discussion

PAL refers to the detection of an air leak for a period exceeding 5-7 days [1]. A fistulous tract causing persistent air leak may be located between the alveoli or bronchioles and the pleural space and is termed an alveolar-pleural fistula (APF) or a bronchopleural fistula (BPF). PAL is most commonly caused by spontaneous pneumothorax, with or without underlying lung disease [1,2]. Among the other causes are pulmonary infections, trauma, thoracic surgery involving lung parenchyma, and mechanical ventilation complications [1].

The quantification of PAL plays an important role in the management of PAL. A three-chamber drainage system is a useful tool for identifying and quantifying air leaks. Recently, Chambers et al. developed a four-grade classification system to determine the severity of air leaks after thoracic surgery [1,2,23,24]. The most severe form of PAL is continuous (C) air leak, which occurs throughout the respiratory cycle. A patient with this type of air leak would require mechanical ventilation with a large underlying BPF [1]. An inspiratory (I) air leak is similarly rare and would require a large fistulous defect in order to occur. Expiratory (E) air leaks are defined as leaks discovered during expiration. The fourth type of leak is found only during forced expiration (FE) or coughing and is known as a forced expiration air leak. However, this system of classification is generally better suited to patients who have undergone thoracic surgery and is not generally applicable to patients who are critically ill [25]. The decision regarding treatment in such patients is usually based on the clinical condition of the patient.

The management of PAL is usually determined by the severity of the treatment and the underlying etiology. The initial assessment is made by sequential balloon inflation and occlusion or injections of methylene blue in order to locate the defect. There are several treatment options available, ranging from conservative management through extended chest tube drainage to chemical and autologous blood patch pleurodesis, endobronchial valve placement, and surgical corrections via video-assisted thoracoscopic surgery (VATS) or open thoracotomy involving mechanical or chemical pleurodesis or pleurectomy [1,2].

A variety of studies have been conducted on autologous blood patches, and they were previously used as a preventative measure for recurrent spontaneous pneumothorax. However, the literature now indicates that it is a safe, effective, and affordable method of treating persistent air leaks caused by pneumothorax [1,2,26-37]. It is believed that autologous blood patches work in two ways. Firstly, the air leak is directly sealed by a blood clot, alongside pleural inflammation caused by blood products. Secondly, the blood clot physically occupies the pleural space, thereby reducing leakage [2].

Ferraroli et al. report a primary study comparing intrapleural fibrin glue with autologous blood patch to surgical thoracotomy. In the study, no statistical significance was found between the two methods, suggesting that a conservative approach with autologous blood patches might reduce the need for surgical intervention and the complications associated with it [7].

According to Chambers et al., they reviewed 10 studies and found that the combined success rate of autologous blood patches is 93%, with 70%-81% of leaks being resolved within 12 hours and 95%-100% within 48 hours [2,30]. In contrast, conventional treatment with chest tube drainage requires 3-6 days of simple tube thoracostomy management [2].

This procedure consists of instilling 50-100 mL of autologous blood through the chest wall into the patient's pleural cavity under sterile conditions [1,2]. Following the flushing and clamping of the tube for around 30-60 minutes, the pleural space is suctioned. Among the most concerning complications of the procedure are tension pneumothorax and infections [2]. It is important to follow up closely with patients before and after the procedure to ensure they are hemodynamically stable and that there is no sign of infection.

Based on the studies included in our review, it appears that ABP is beneficial in the setting of PAL. Ibrahim et al. reported a mean time to seal PAL to be five days as opposed to 10 days in the observation group [14]. Lillegard et al. found that five out of eight patients improved almost instantly, with one patient improving on day 1 and two patients improving on day 2 [28]. There does not seem to be an obvious relation with the dose of autologous blood administered, as evidenced by a study reported by Cao et al., which reports a rather unpredictable pattern to improvement in PAL after the administration of different doses of autologous blood patches [12].

A meta-analysis was conducted during this study in order to evaluate the clinical outcomes and complications associated with the treatment of PAL with autologous blood patches. During the meta-analysis, we observed a clinically significant reduction in air leak repair times in patients who received autologous blood patches, as compared with our control group, as reported by Andreetti et al. [11], Ibrahim et al. [14], and Zhang et al. [13] (Figure 2 and Figure 3). Furthermore, there was no clinical difference between 50 mL of autologous blood and 100 mL of blood used during the procedure, as reported in the meta-analysis of the studies reported by Akar et al. [6] and Andreetti et al. [11] (Figure 4 and Figure 5).

We noted that patients who received the treatment with autologous blood patch did not show any statistically significant decrease in the time to discharge as opposed to the control group, which was reported in the meta-analysis of the studies of Cagirici et al. [20], Ibrahim et al. [14], and Zhang et al. [13] (Figure 6 and Figure 7). Data for infection, fever, and pain was evaluated as complications of the treatment with autologous blood patch. The meta-analysis performed on the available data shows no statistical significance between the experimental and control arm in terms of infection rates and pain (Figure 8 and Figure 9); however, it does show significance in terms of fever in the experimental group (Figure 8 and Figure 9) [11-16].

A literature review also shows an improvement in PAL in children, as well as improved clinical outcomes [28,29]. Current evidence indicates that ABP may be considered a gold standard or first-line treatment for PAL caused by certain medical conditions, such as acute respiratory distress syndrome (ARDS) and ILD [37]. However, a lack of RCT data may be viewed as a potential disadvantage. It is necessary to collect more data through RCTs in order to quantify the potential magnitude of benefits associated with ABP.

## Conclusions

Recent data regarding the treatment of persistent air leaks with autologous blood patches indicates promising results that suggest that the conservative management of persistent air leaks with autologous blood patches may be comparable in treatment efficacy and outcome without surgical intervention. Further research is required, however, in order to fully assess the non-inferiority of autologous blood patches in comparison with surgical treatment and other conventional treatments.

## References

[1] Dugan KC, Laxmanan B, Murgu S, Hogarth DK (2017). Management of persistent air leaks. Chest.

[2] Sakata KK, Reisenauer JS, Kern RM, Mullon JJ (2018). Persistent air leak - review. Respir Med.

[3] Moher D, Liberati A, Tetzlaff J, Altman DG (2009). Preferred reporting items for systematic reviews and meta-analyses: the PRISMA statement. Ann Intern Med.

[4] National Heart, Lung Lung, and Blood Institute. (2014 (2014). National Heart, Lung, and Blood Institute: study quality assessment tools. https://www.nhlbi.nih.gov/health-topics/study-quality-assessment-tools.

[5] Apilioğulları B, Dumanlı A, Ceran S (2020). Application of autologous blood patch in patients with non-expanded lungs and persistent air leak. Turk Gogus Kalp Damar Cerrahisi Derg.

[6] Akar E, Haberal MA, Şengören Dikiş Ö (2020). The effectiveness of blood amount used in pleurodesis to prevent prolonged air leakage. Turk Gogus Kalp Damar Cerrahisi Derg.

[7] Ferraroli GM, Perroni G, Giudici VM (2022). Bronchoscopic intra-pleural instillation of fibrin glue and autologous blood to manage persistent air leaks after lung resection. J Clin Med.

[8] Dye K, Jacob S, Ali M, Orlando D, Thomas M (2020). Autologous blood patching to mitigate persistent air leaks following pulmonary resection: a novel approach. Cureus.

[9] Aihara K, Handa T, Nagai S (2011). Efficacy of blood-patch pleurodesis for secondary spontaneous pneumothorax in interstitial lung disease. Intern Med.

[10] Evman S, Alpay L, Metin S (2016). The efficacy and economical benefits of blood patch pleurodesis in secondary spontaneous pneumothorax patients. Kardiochir Torakochirurgia Pol.

[11] Andreetti C, Venuta F, Anile M (2007). Pleurodesis with an autologous blood patch to prevent persistent air leaks after lobectomy. J Thorac Cardiovasc Surg.

[12] Cao GQ, Kang J, Wang F, Wang H (2012). Intrapleural instillation of autologous blood for persistent air leak in spontaneous pneumothorax in patients with advanced chronic obstructive pulmonary disease. Ann Thorac Surg.

[13] Zhang HT, Xie YH, Gu X (2019). Management of persistent air leaks using endobronchial autologous blood patch and spigot occlusion: a multicentre randomized controlled trial in China. Respiration.

[14] Ibrahim IM, Elaziz ME, El-Hag-Aly MA (2019). Early autologous blood-patch pleurodesis versus conservative management for treatment of secondary spontaneous pneumothorax. Thorac Cardiovasc Surg.

[15] Shackcloth MJ, Poullis M, Jackson M, Soorae A, Page RD (2006). Intrapleural instillation of autologous blood in the treatment of prolonged air leak after lobectomy: a prospective randomized controlled trial. Ann Thorac Surg.

[16] Khan MA, Bhat MA, Majeed A (2017). Intrapleural therapy for the prevention of recurrent spontaneous pneumothorax-a randomized comparative evaluation of bleomycin pleurodesis & autologous blood pleurodesis. Int J Adv Res Ideas Innov Tech.

[17] Narenchandra V, Vishnukanth G, Dwivedi DP (2022). Comparison of efficacy of autologous blood patch pleurodesis versus doxycycline pleurodesis in the management of persistent air leak in patients with secondary spontaneous pneumothorax. A randomized control trial. Monaldi Arch Chest Dis.

[18] Martínez-Escobar S, Ruiz-Bailén M, Lorente-Acosta MJ, Vicente-Rull JR, Martínez-Coronel JF, Rodríguez-Cuartero A (2006). Pleurodesis using autologous blood: a new concept in the management of persistent air leak in acute respiratory distress syndrome. J Crit Care.

[19] Ando M, Yamamoto M, Kitagawa C (1999). Autologous blood-patch pleurodesis for secondary spontaneous pneumothorax with persistent air leak. Respir Med.

[20] Cagirici U, Sahin B, Cakan A, Kayabas H, Buduneli T (1998). Autologous blood patch pleurodesis in spontaneous pneumothorax with persistent air leak. Scand Cardiovasc J.

[21] Cobanoglu U, Melek M, Edirne Y (2009). Autologous blood pleurodesis: a good choice in patients with persistent air leak. Ann Thorac Med.

[22] Lang-Lazdunski L, Coonar AS (2004). A prospective study of autologous 'blood patch' pleurodesis for persistent air leak after pulmonary resection. Eur J Cardiothorac Surg.

[23] Cerfolio RJ, Bass C, Katholi CR (2001). Prospective randomized trial compares suction versus water seal for air leaks. Ann Thorac Surg.

[24] Cerfolio RJ, Tummala RP, Holman WL (1998). A prospective algorithm for the management of air leaks after pulmonary resection. Ann Thorac Surg.

[25] Kurman JS (2021). Persistent air leak management in critically ill patients. J Thorac Dis.

[26] Oliveira FH, Cataneo DC, Ruiz RL Jr, Cataneo AJ (2010). Persistent pleuropulmonary air leak treated with autologous blood: results from a university hospital and review of literature. Respiration.

[27] Robinson CL (1987). Autologous blood for pleurodesis in recurrent and chronic spontaneous pneumothorax. Can J Surg.

[28] Lillegard JB, Kennedy RD, Ishitani MB, Zarroug AE, Feltis B (2013). Autologous blood patch for persistent air leak in children. J Pediatr Surg.

[29] Pruitt LC, Kastenberg ZJ, Fenton SJ, Short SS (2021). Early use of autologous blood patch pleurodesis in children is successful in resolving persistent air leaks. J Pediatr Surg.

[30] Chambers A, Routledge T, Billè A, Scarci M (2010). Is blood pleurodesis effective for determining the cessation of persistent air leak?. Interact Cardiovasc Thorac Surg.

[31] Ploenes T, Kyritsis I, Mardanzai K (2020). A prospective study investigating blood patch pleurodesis for postoperative air leaks after pulmonary resection. J Surg Res.

[32] Li BZ, Zhang XG, Li WQ, Li ZT, Guo HQ, Jiang FS (2021). [Pleurodesis with an Autologous Blood Patch in the Treatment of Persistent Air Leak after Lung Resection] [Article in Chinese]. Zhongguo Yi Xue Ke Xue Yuan Xue Bao.

[33] Campisi A, Dell'Amore A, Gabryel P (2022). Autologous blood patch pleurodesis: a large retrospective multicenter cohort study. Ann Thorac Surg.

[34] Hasan IS, Allen MS, Cassivi SD (2021). Autologous blood patch pleurodesis for prolonged postoperative air leaks. J Thorac Dis.

[35] Droghetti A, Schiavini A, Muriana P (2006). Autologous blood patch in persistent air leaks after pulmonary resection. J Thorac Cardiovasc Surg.

[36] Shaw JA, Wilken E, Allwood BW, Irusen EM, Koegelenberg CF (2022). Autologous blood patch pleurodesis for the management of a persistent air leak after secondary spontaneous pneumothorax. Respiration.

[37] Manley K, Coonar A, Wells F, Scarci M (2012). Blood patch for persistent air leak: a review of the current literature. Curr Opin Pulm Med.

